# Deuterium magnetic resonance imaging of tumors using low-dose systemic deuterated water labeling

**DOI:** 10.3389/fonc.2025.1688968

**Published:** 2026-01-12

**Authors:** Mingchen He, Jill M. Duzen, Terri Larus, Hunter Reavis, Steven G. Turowski, Michael E. Feigin, Don E. Farthing, Joseph A. Spernyak, Nataliya Prokopenko Buxbaum

**Affiliations:** 1 Department of Pediatrics, Roswell Park Comprehensive Cancer Center, Buffalo, NY, United States; 2 Center for Immuno-Oncology, Center for Cancer Research, National Cancer Institute, National Institutes of Health, Bethesda, MD, United States; 3 Department of Pharmacology and Therapeutics, Roswell Park Comprehensive Cancer Center, Buffalo, NY, United States; 4 Department of Cell Stress Biology, Roswell Park Comprehensive Cancer Center, Buffalo, NY, United States

**Keywords:** cancer, deuterated water, deuterium oxide, isotope labeling, magnetic resonance imaging, neoplasms, mass spectrometry

## Abstract

**Background:**

Although systemic ^2^H_2_O labeling is an attractive clinically relevant strategy for enhancing tumor imaging given relative ease of administration, gradual, slow ramp-up of labeling is preferable to avoid side effects, such as nausea and dizziness. Slow labeling periods are more easily tolerated but are suboptimal in the setting of cancer given the need to promptly diagnose and stage patients to initiate treatment. Hence, we sought to deploy low systemic concentrations of ^2^H_2_O in total body water (TBW) with short durations and low magnetic field strength to test clinically relevant experimental parameters conducive to clinical translation.

**Methods:**

HT-29 cells and KPC cells were used to establish subcutaneous and orthotopic murine tumor models, respectively. Systemic ^2^H_2_O administration was performed and TBW enrichment was confirmed via urine testing. All mice were imaged using the 7T MRI with a dual tuned (^1^H/^2^H) leg or body coil after 1, 3, or 7 days of systemic ^2^H_2_O labeling to 2 or 4% TBW (v/v).

**Results:**

The concentrations of ^2^H_2_O in urine samples closely reflected the target ^2^H_2_O enrichment level specified in the systemic labeling protocol. In all tested experimental schemas, there were no statistically significant differences in tumor-muscle contrast-to-noise ratio (CNR) and signal ratio among different labeling durations (*p* > 0.05), despite a significantly higher CNR at 4% compared with 2% (*p* = 0.02).

**Conclusion:**

Reasonable and stable imaging contrast between tumor and healthy tissue during the systemic labeling was achieved in this pre-clinical study, which offer preliminary indications of potential clinical translation of this imaging approach in cancer.

## Introduction

Positron emission tomography (PET) is a widely used clinical imaging modality to evaluate metabolic features of cancer, stage disease, and measure treatment responses. PET results in ionizing radiation exposure of patients, beyond levels received from computerized tomography typically used for anatomical referencing with the former. For cancer patients, especially treated in childhood and adolescence, those with higher potential for cure and therefore long-term survival, radiation exposure from medical imaging contributes to latent secondary malignancies (1, 2).

Deuterium magnetic resonance imaging (dMRI) has recently emerged as a promising tool for metabolic tumor imaging (3, 4). Aside from eliminating exposure to ionizing radiation associated with PET, additional metabolic features of cancer, i.e., glycolytic rates, which provide biologically relevant insight into tumor biology *in situ*, can be evaluated by dMRI. Previous studies mostly used glucose as the deuterium labeled substrate (5) to measure downstream metabolites or kinetics of glucose turnover, but such protocols typically require prolonged scan times to qualify dynamic metabolic changes based on the known natural abundance of deuterium (6). For example, in tumor imaging studies, the lactate peak is usually most prominent an hour after administration of d-glucose (7). Furthermore, low signal-to-noise ratio (SNR) continues to pose a significant barrier to widespread application of this approach, particularly at field strengths of 3T and below, which are typically used in clinical settings (8). Currently, most studies are limited to ultra-high field MRI (9–11). Additionally, deuterated glucose administration may not be appropriate for diabetic patients or those on GLP-1 receptor agonists; hence, our institutional criteria for enrollment on dMRI studies currently exclude such patients. Meanwhile, deuterated water (^2^H_2_O) is easier to administer, cheaper, and has been safely used in clinical studies (12), including in pediatric patients. Therefore, we focused on deuterium imaging signal differences between tumor and healthy normal tissue using clinically relevant systemic ^2^H_2_O labeling doses and pre-clinical dMRI (13) at field strength (7T) that inform clinical imaging potential at 3T, and aimed to complement existing research methods by providing an additional tool through this technique.

Analogous to the heightened glucose demand of tumors (14), tumors tend to have higher water content compared to normal tissues of similar ontogeny as measured by near-infrared FT Raman spectroscopy (15). Our previous imaging studies showed that tumor regions and GvHD-affected tissues infiltrated by proliferating activated T cells exhibited higher deuterium signals than normal tissues after ^2^H_2_O systemic labeling, and deuterium MR spectroscopy indicated that these elevated signals were predominantly driven by the water peak (13, 16). Thus, systemic administration of ^2^H_2_O results in greater intracellular deuterium content within proliferating compared to quiescent cells, which can be detected by dMRI. We previously showed excellent deuterium imaging contrast between tumor xenografts and muscle at 9.4T with 7 days of 8% ^2^H_2_O in total body water (TBW), v/v (13). Although ^2^H_2_O is considered relatively safe to ingest, the cost and time needed to gradually label a person to such concentrations of ^2^H_2_O is not practical in clinical studies. Rapid ramp ups can be performed (17), but would warrant intravenous rather than PO labeling and have higher propensity for causing unwanted side effects of rapidly increasing ^2^H_2_O in TBW, i.e., vertigo and nausea (18, 19). Systemic labeling with ^2^H_2_O to 1-2% TBW, v/v, has been safely performed for over a decade in people, including children (12, 17, 20). Therefore, to facilitate clinical translation of our labeling-imaging approach for tumor assessment, we tested lower TBW levels and shorter duration of systemic labeling and performed pre-clinical animal imaging at a lower magnetic field, 7T, which suggests feasibility of imaging humans using this approach at 3T. While small rodent imaging studies conducted at 7T cannot fully predict clinical utility at 3T due to the expected reduction of net magnetization vector and negative impact of larger coil diameter, other factors could mitigate signal reduction, including significantly larger voxel sizes used for imaging patients, much greater body mass (~3500-fold), the possibility of using quadrature or surface or array coils, and shorter T1 and longer T2 times at lower field strengths (21). Thus, we are leveraging pre-clinical data provided herein in planning early phase clinical trials, which will ultimately inform the field on utility and feasibility of this approach in the clinic.

## Materials and methods

### Mice and cells

All animal experiments were performed in accordance with Roswell Park Comprehensive Cancer Center (RPCCC)’s approved IACUC protocol. Nine female SCID mice 12-weeks of age were obtained from the RPCCC on-site breeding facility, for the xenograft study. HT-29, a human colon adenocarcinoma cell line with adherent growth characteristics, was purchased from ATCC (cat# HTB-38). Approximately 2 × 10^6^ HT-29 cells suspended in 100 µL PBS were subcutaneously injected into proximal hind limb of each SCID mouse with contralateral hind limb left unmanipulated (control). Imaging commenced 8 days after tumor implantation.

Five female C57BL/6 mice aged 8–10 weeks were purchased from The Jackson Laboratory for the orthotopic study. KPC (LSL-KRAS^G12D/+^; LSL-TP53^R172H/+^; Pdx-1-Cre) cells, mouse pancreatic ductal adenocarcinoma cancer cell line (sex-matched), KRAS model, were obtained from the Feigin lab, RPCCC. Syngeneic orthotopic grafts were surgically implanted into the pancreas. Approximately 10,000 KPC syngeneic cells suspended in 20uL of DMEM supplemented with antibiotic-free serum were orthotopically injected into the pancreas. The pancreas was then re-internalized, and the peritoneal cavity was closed using 5–0 Vicryl sutures. Wound clips were used to close the skin and were removed 10 days post-op. Animals were monitored and evaluated daily after surgery. Imaging commenced 14 days after tumor implantation.

### Systemic deuterium labeling

**Figure 1:**
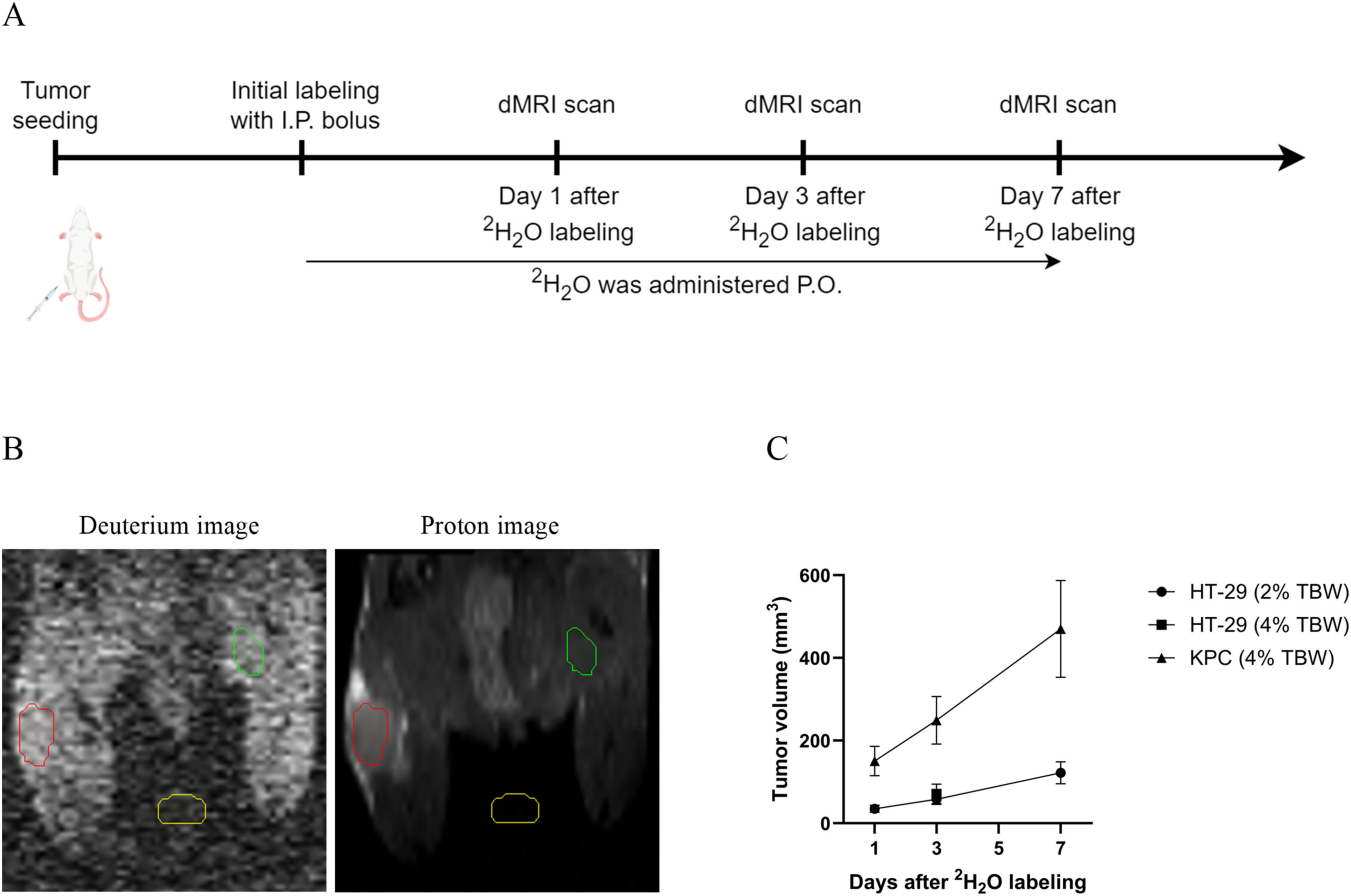
**(A)** Experimental schema of systemic labeling and imaging protocols. An initial bolus injection was given to the mice, then deuterated drinking water ad lib for the duration of the experiment to maintain target ^2^H_2_O enrichment levels in TBW was provided. **(B)** Representative 3D-FLASH coronal deuterium image and its corresponding proton image of tumor-bearing mouse with 2% ^2^H_2_O enrichment in TBW (v/v), with regions of interest outlined in different colors (red: tumor; green: contralateral limb muscle; yellow: air). **(C)** Tumor volume during the loading period in the two models.

A 35 µL/g body weight bolus containing 0.9% NaCl in 100% ^2^H_2_O (Cambridge Isotope Laboratories) was administered via intraperitoneal (IP) injection to achieve rapid 4% TBW enrichment. The mice were then provided 8% deuterated drinking water (v/v) *ad lib* for the duration of the experiment to maintain 4% ^2^H_2_O in TBW to account for the expected ~30% ^2^H_2_O losses from respiration and perspiration (13, 17) (**Figure 1A**). A 17 µL/g body weight IP bolus was used to achieve a rapid 2% TBW enrichment, followed by *ad lib* supplementation of 4% deuterated drinking water (v/v) for the duration of the experiment to maintain 2% ^2^H_2_O in TBW (v/v).

### Urine TBW deuterium enrichment measurement

For a subset of mice labeled to 2% ^2^H_2_O enrichment in TBW, urine sampling was performed via spontaneous passage to confirm that TBW goal had been achieved. 25-100µL urine was collected into an Eppendorf microcentrifuge tube for each mouse and stored at -80°C until head space gas chromatography-negative chemical ionization mass spectrometry (HS-GC-NCI-MS) assay was performed, as previously described (22).

### MRI

MRI studies were performed on a 7T Bruker Biospin animal scanner with custom-made, dual tuned (^1^H/^2^H) Tx/Rx leg and body volume coils. A ^1^H coronal, 3D gradient echo scan was acquired for anatomical reference, as well as T2-weighted, multi-slice spin-echo scans in the axial and coronal orientations to assist in tumor localization. Detailed scan parameters can be found in the MR Acquisition Parameters section of the Supplemental Information, **Supplementary Table S1**. Deuterium image acquisition was performed using a 3D, fast low angle shot (FLASH) sequence (TR/TE=100/4 ms; flip angle=30; averages=2 (subcutaneous xenograft), 8 (orthotopic allograft), respectively; FOV = 48 × 32 × 32 mm^3^; matrix size: subcutaneous 128 × 64 × 64, orthotopic 96 × 32 × 32; acquisition time=15 minutes). Both 3D scans (^1^H/^2^H) were acquired with identical geometries to simplify co-registration.

### MRI image analysis

All images were loaded into Analyze 14.0 for registration and analysis. Following spatial co-registration of ^1^H scans to the ^2^H acquisition, details can be found in the MRI Co-registration Workflow section of the Supplemental Information, **Supplementary Figure S1**; regions of interest (ROIs) were drawn manually in tumor, muscle and air, **Figure 1B**. SNR, the contrast-to-noise ratio (CNR) and tumor-to-muscle deuterium signal ratio (T/M ratio) were calculated based on the image intensity distributions. SNR = SI_tumor_/SD_air_; CNR = (SI_tumor_ – SI_muscle_)/SD_air_; T/M ratio = SI_tumor_/SI_muscle_. SI_tumor_ represents the mean signal intensity of the tumor, SI_muscle_ denotes the mean signal intensity of the muscle, and SD_air_ represents the standard deviation of the background noise. A low-pass filter with a 3×3×3 kernel was applied to the deuterium images displayed.

### Statistical analysis

Differences in SNR and CNR between cohorts with the two different levels of ^2^H_2_O enrichment in TBW were assessed by the Mann-Whitney U-test. To assess whether the tumor–muscle CNR differed significantly from zero, one-sample t-tests were conducted for both models. Differences in ^2^H_2_O concentrations in urine, SNR, CNR and tumor-to-muscle deuterium signal ratio (T/M ratio) among groups with different labeling durations were analyzed by one-way repeated measures ANOVA. Accordingly, *post hoc* analyses using Tukey’s multiple comparisons test were performed. All multiple comparisons were adjusted to control the family-wise error rate. Simple linear regression was used to estimate the relationship between ^2^H_2_O concentration in urine and SNR in tumor. Group comparisons used two-sided statistical tests. Results were considered significant when p-values were less than 0.05. Data analysis and visualization were performed with GraphPad Prism 10.4.1. Descriptive data are presented as mean ± standard deviation, with 95% confidence intervals reported where applicable. Graphs depict the mean values with standard error of the mean.

## Results

Tumor volumes increased gradually across the study period in both models. In the HT-29 model, tumor volumes were 35.48 ± 26.90 mm^3^ on Day 1, 58.25 ± 38.01 mm^3^ on Day 3, and 122.1 ± 80.15 mm^3^ on Day 7 for the 2% TBW group; the 4% TBW group was included for Day 3 only, with a tumor volume of 71.10 ± 58.02 mm^3^. In the KPC model, tumor volumes increasing from 150.9 ± 79.32 mm^3^ on Day 1 to 249.3 ± 129.2 mm^3^ on Day 3 and 470.1 ± 261.6 mm^3^ on Day 7, **Figure 1C**.

### Urine TBW deuterium enrichment

**Figure 2:**
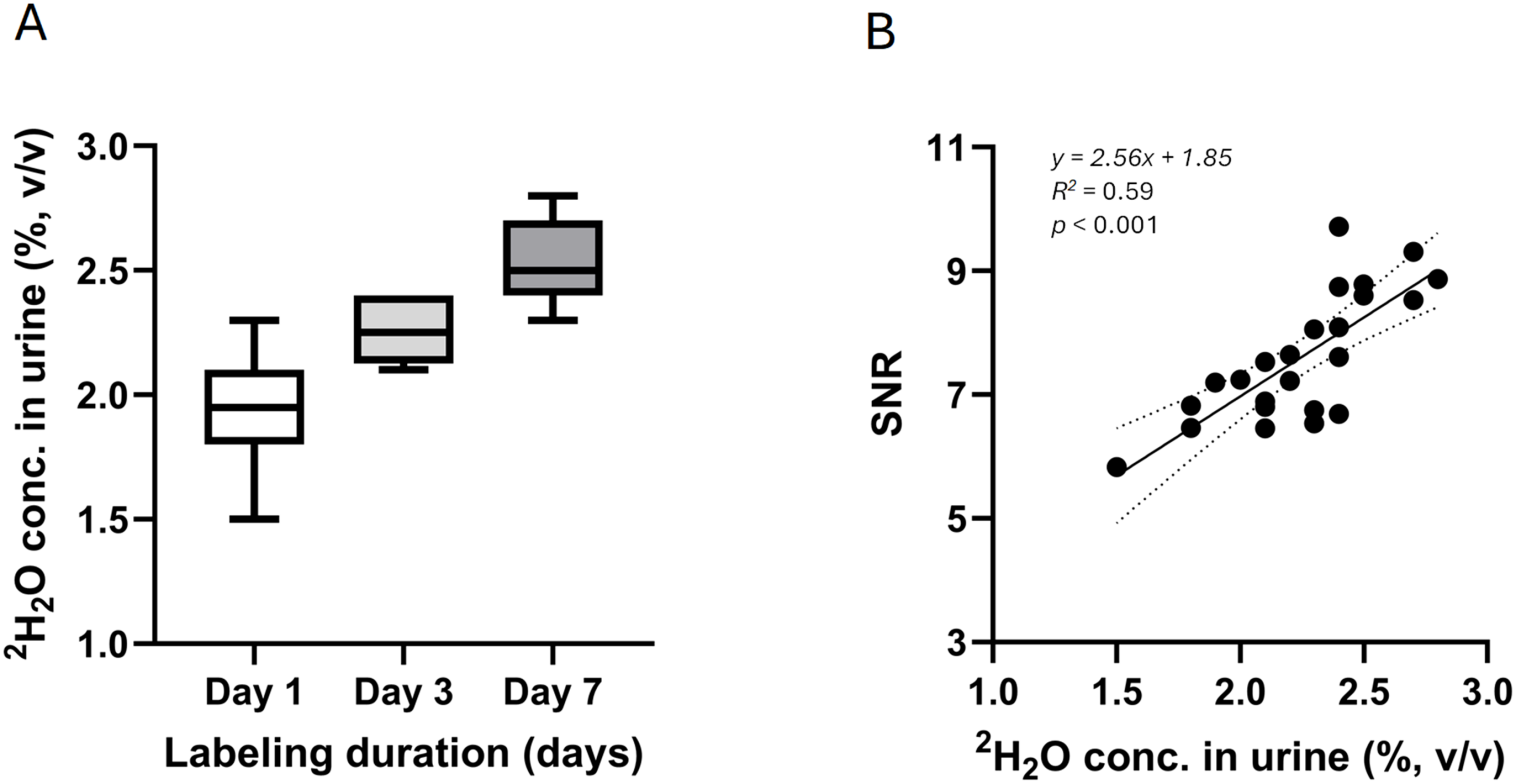
**(A)**^2^H_2_O concentrations in urine were assessed for different labeling duration within the 2% ^2^H_2_O TBW cohort for subcutaneous HT-29 xenograft model, and progressive accumulation of ^2^H_2_O was observed, with intergroup variation. **(B)** The scatter plot showed a positive relationship between ^2^H_2_O concentration in urine/total body water (TBW) and ^2^H imaging SNR in tumor.

^2^H_2_O concentrations in urine samples were comparable to the target ^2^H_2_O enrichment level of 2% specified in the systemic labeling protocol for the subcutaneous xenograft model, though progressive accumulation of ^2^H_2_O from 1.94 ± 0.24 at Day 1 to 2.26 ± 0.13 at Day 3 and 2.54 ± 0.18 at Day 7 (all in % v/v) was observed during the labeling, with statistically significant differences across the three groups (Day 1 vs. Day 3, *p* = 0.01; Day 1 vs. Day 7, *p* = 0.004; Day 3 vs. Day 7, *p* = 0.01), **Figure 2A**. Linear regression analysis revealed a significant positive relationship between ^2^H_2_O concentration in urine and SNR in tumor (*p* < 0.001), with a moderately strong linear correlation (*R²* = 0.59), **Figure 2B**.

### HT-29 subcutaneous xenograft model

**Figure 3:**
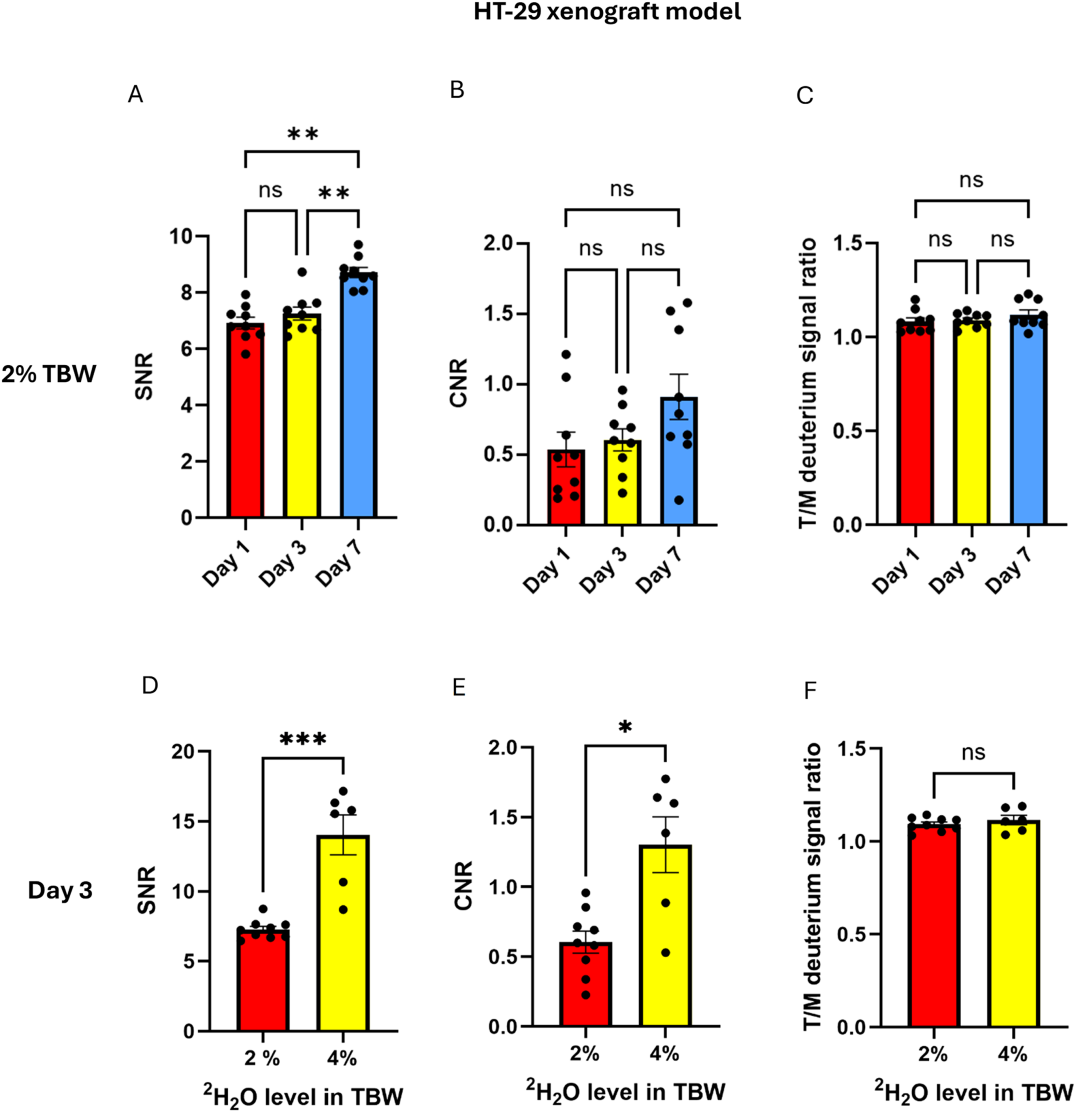
Comparison of SNR, CNR and T/M ratios in the HT-29 xenograft model across different labeling durations and ^2^H_2_O TBW levels. **(A–C)** SNR, CNR and T/M ratios were compared across different labeling durations within the 2% ^2^H_2_O TBW cohort. **(D–F)** SNR, CNR and T/M ratios were compared between 2% and 4% ^2^H_2_O in TBW cohorts on day 3 of continuous labeling. ns: *p* > 0.05; *p < 0.05; **p < 0.01; ***p < 0.001.

**Figure 4:**
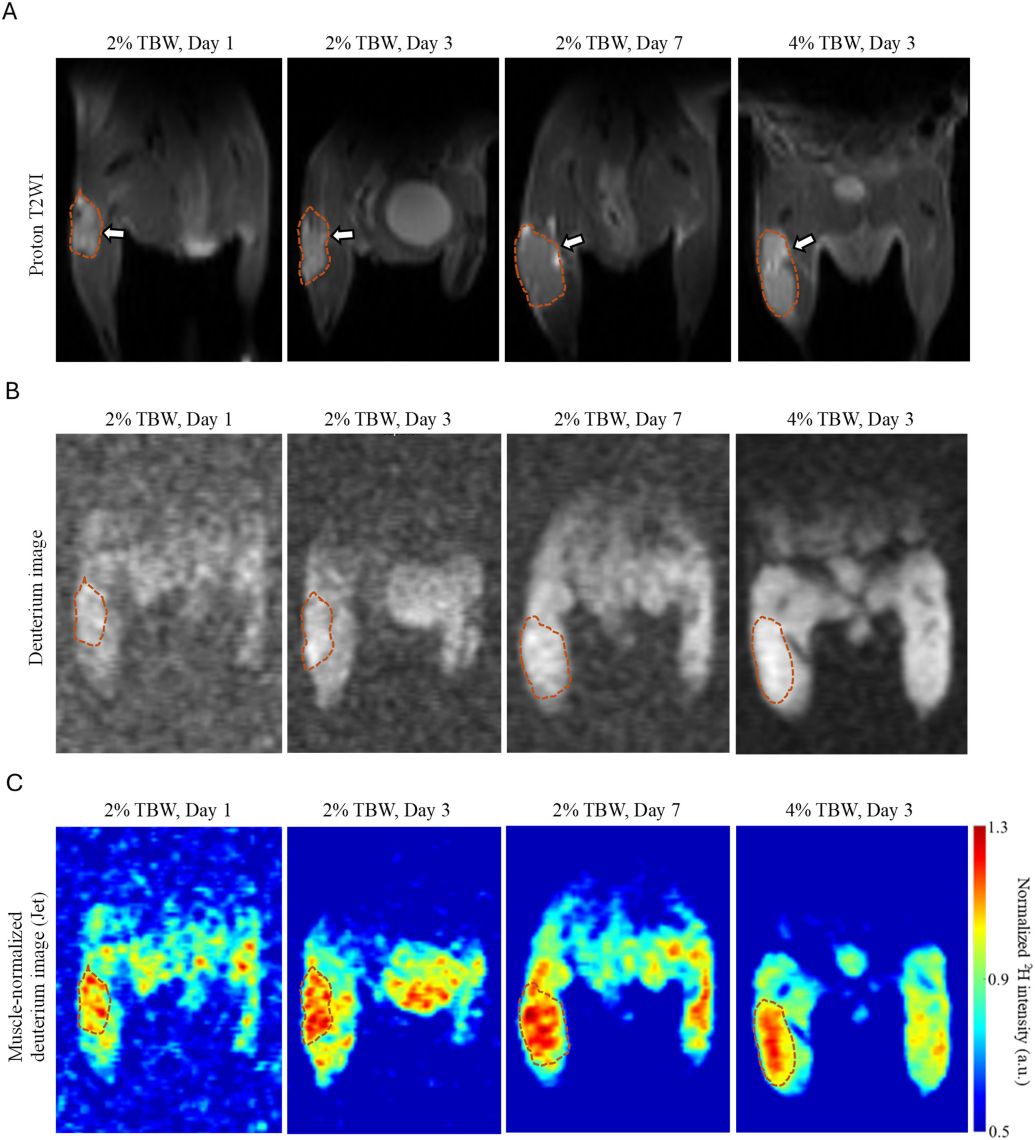
Representative images from the HT-29 xenograft model comparing different labeling durations and concentrations. **(A)** Coronal proton T2-weighted images: the arrows point to subcutaneous tumors delineated with dashed lines in the hind limb. **(B)** Aligned deuterium images of **(A)**. **(C)** Corresponding muscle-normalized deuterium images of B did not show significant differences in signal intensity across different loading days and loading percentages (4% vs. 2%, Day 3), displayed using “Jet” color scale.

Effect of loading days (2% cohort): The SNR at Day 7 (8.72 ± 0.63) was significantly higher than at Day 1 (6.31 ± 0.63, *p* = 0.002) and Day 3 (7.27 ± 0.69, *p* = 0.002), **Figure 3A**. The mean tumor–muscle CNR values at Day 1, Day 3 and Day 7 were 0.55 (95% CI: 0.25–0.82, *p* = 0.003), 0.60 (95% CI: 0.42–0.78, *p* < 0.001), and 0.91 (95% CI: 0.54–1.28, *p* < 0.001). One-sample t-tests indicated significant tumor–muscle contrast at all-time points, whereas one-way repeated measures ANOVA showed no statistically significant differences in CNR (*F* (1.530, 12.24) = 2.399, *p* = 0.14) between the three labeling duration schemas, **Figure 3B**. No significant differences in T/M ratio were observed between groups with different labeling durations (*F*(1.524, 12.19) = 0.957, *p* = 0.39) **Figures 3C**, **4**.

Effect of loading percentage (4% vs. 2%, Day 3): Higher ^2^H_2_O level in TBW resulted in statistically significant higher SNR (14.03 ± 3.47 vs. 7.27 ± 0.69, *p* < 0.001), **Figure 3D**. CNR between 4% and 2% ^2^H_2_O TBW level was statistically significant as well, with the former being higher (1.30 ± 0.49 vs. 0.60 ± 0.23, *p* = 0.02), **Figure 3E**. No significant differences in T/M ratio were observed between these two concentration-defined cohorts (*p* = 0.61), **Figures 3F**, **4**.

### KPC syngeneic orthotopic model

**Figure 5:**
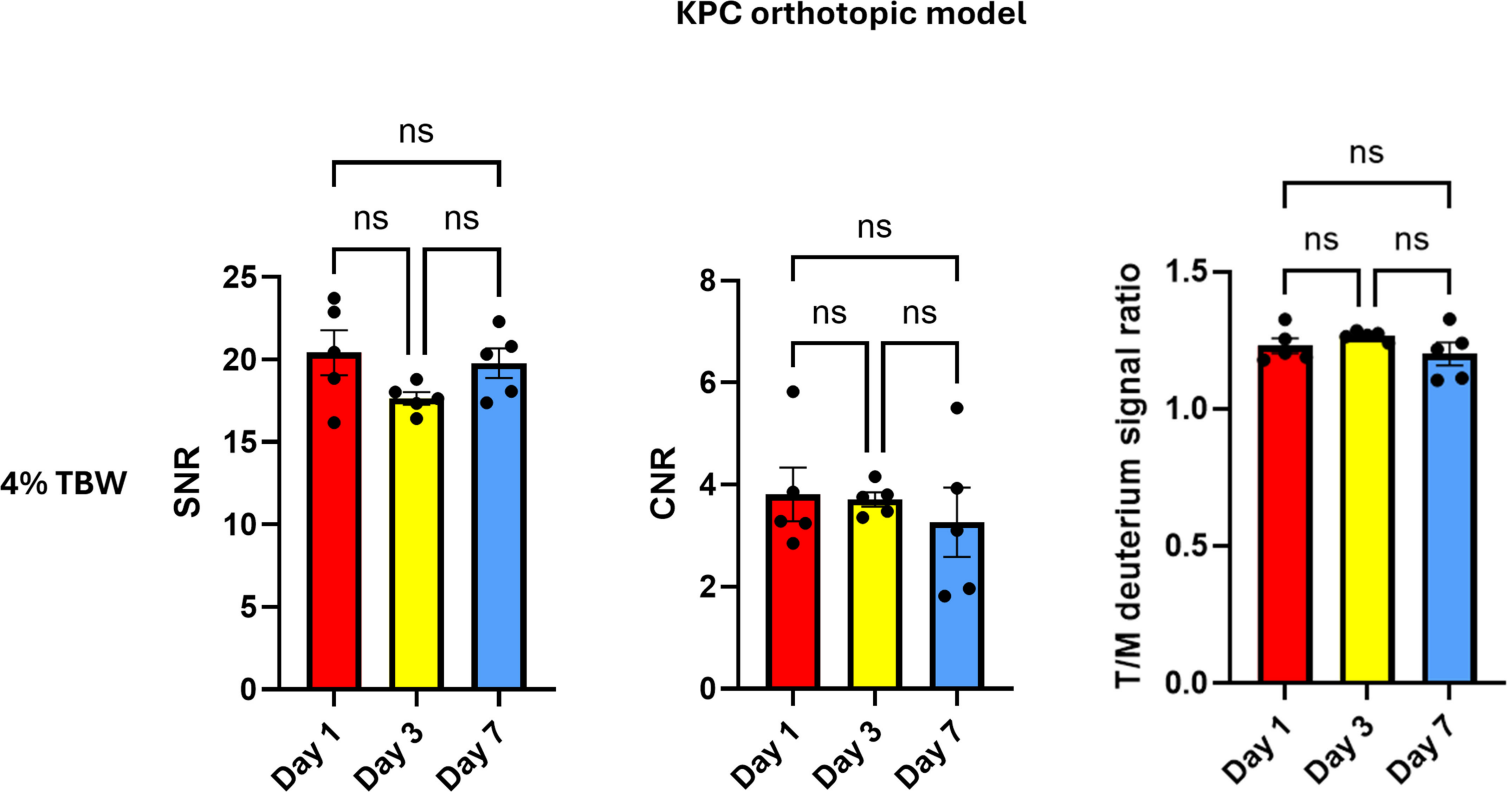
Comparison of SNR, CNR and T/M ratios in the KPC orthotopic model across different ^2^H_2_O labeling durations at ~4% ^2^H_2_O in TBW. No statistically significant differences were observed. ns: *p* > 0.05.

**Figure 6:**
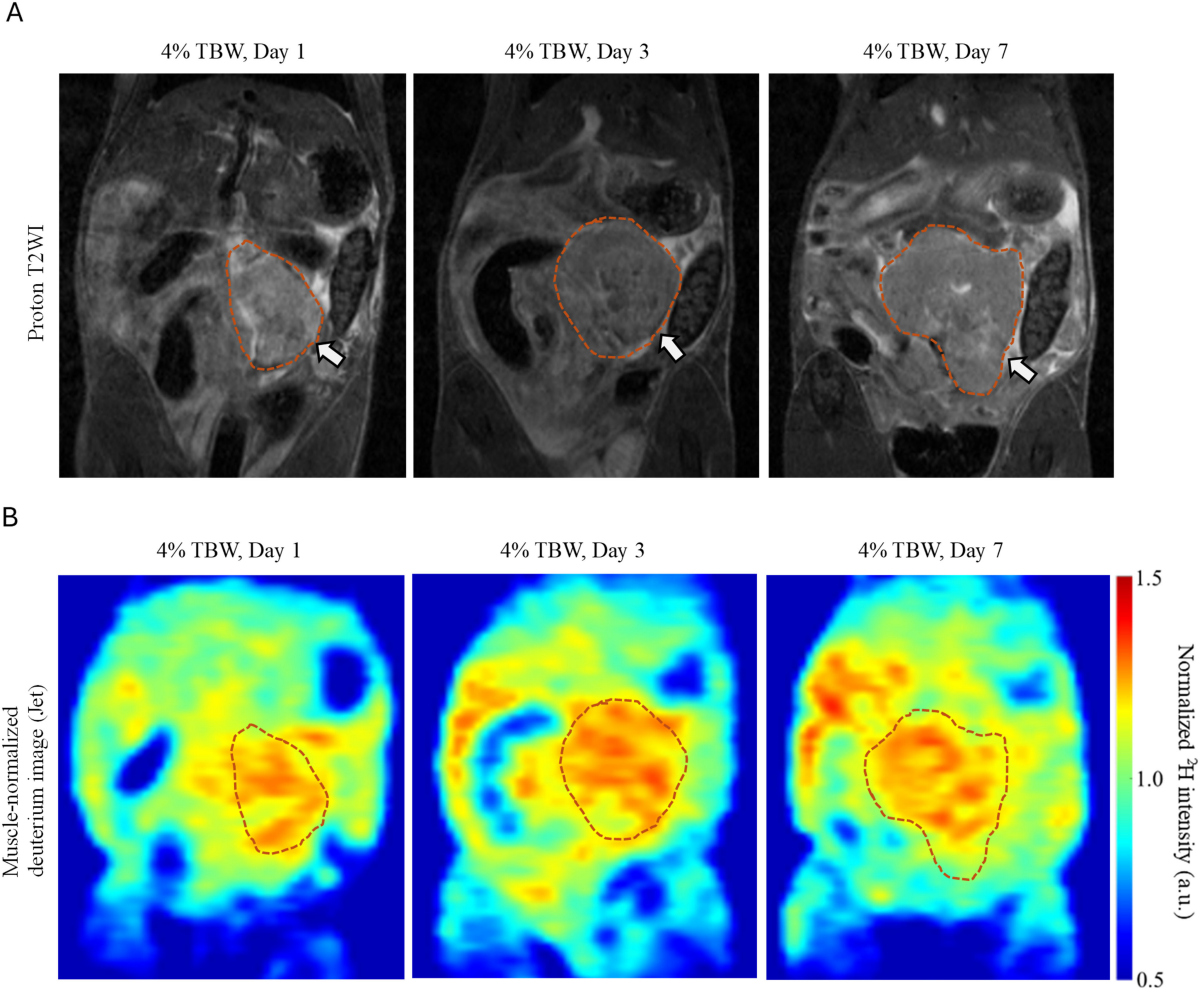
Representative images from the KPC orthotopic model comparing different labeling durations at ~4% ^2^H_2_O in TBW. **(A)** Coronal proton T2-weighted images: the arrows point to orthotopic tumors delineated with dashed lines in the pancreas. **(B)** Corresponding muscle-normalized deuterium images did not show significant differences in signal intensity across different loading days, displayed using “Jet” color scale.

The SNR at Day 1 (20.42 ± 3.06) was not statistically significant different from those at Day 3 (17.65 ± 0.88, *p* = 0.11) or Day 7 (19.78 ± 2.02, *p* = 0.64). The difference between Day 3 and Day 7 also did not reach significance (*p* = 0.06). The deuterium signal intensities of tumor tissues were higher than those of normal muscle tissues. The mean tumor–muscle CNR values at Day 1, Day 3 and Day 7 were 3.81 (95% CI: 2.34–5.28, *p* = 0.002), 3.71 (95% CI: 3.32–4.10, *p* < 0.001) and 3.26 (95% CI: 1.37–5.15, *p* = 0.009). One-sample t-tests indicated significant tumor–muscle contrast at all-time points; while no statistically significant differences were observed in CNR (*F*(1.213, 4.853) = 0.855, *p* = 0.42) and T/M ratio (*F*(1.306, 5.224) = 2.664, *p* = 0.16) across the three different labeling durations, **Figures 5**, **6**.

## Discussion

In the current pre-clinical study, we tested clinically relevant systemic ^2^H_2_O TBW labeling schemas and confirmed that targeted ^2^H_2_O concentrations in TBW were indeed reached via urine sample TBW enrichment measurements. In our subcutaneous xenograft model, we showed that 4% ^2^H_2_O in TBW (v/v) and longer duration of labeling (7 days) demonstrated higher SNR at 7T compared to labeling to 2% TBW with shorter duration; however, imaging contrast was not enhanced with higher and longer duration of labeling with no significant difference in T/M ratios among different groups, which the latter being a parameter that is most clinically meaningful. Notably, in our orthotopic model, SNR, CNR and T/M ratio did not differ significantly among groups with different labeling durations, reinforcing that lower concentrations and durations of labeling that are more conducive to clinical translation would generate sufficient imaging tumor contrast.

In current clinical practice, traditional proton anatomical imaging has provided high-quality images with high SNR, while deuterium imaging may serve as a potentially useful complement to proton anatomical imaging, offering metabolic insight into tumor tissue. In this regard, enhancing the signal contrast between tumor tissue and normal tissue is crucial. An additional consideration as we translate this approach into the clinic is that performing longitudinal comparisons of standardized images may be more meaningful for evaluation of treatment effectiveness. Based on this, we normalized tumor signal to muscle and compared the T/M ratios across groups finding no statistically significant differences among them. In contrast, the orthotopic model did not exhibit significant differences in SNR among groups with different labeling durations, as well as in CNR and T/M ratio. This may be due to the biological differences between the two models; for example, HT-29 tumors exhibited a shorter volume-doubling time, suggesting a faster relative volumetric growth rate which may help to explain the increase in CNR at Day 7 observed within HT-29 tumors but not KPC. It is worth noting that the leg volume coil demonstrated a 3.4-fold higher SNR compared to the body volume coil under identical scanning conditions (**Supplementary Figure S2**). Hence, a limitation of our study is that we used two different coils to acquire data from two different tumor models, which may have introduced variability, thus limiting our ability to directly compare the two datasets. However, we have shown that systemic labeling showed positive and visible contrast between tumor and normal tissue using two distinct backgrounds and implantation sites, indicating the technique’s potential translatability to multiple regions/anatomical locations. Furthermore, improved conspicuity of tumors from normal tissue can be achieved in future studies with only minor reductions in acquisition matrix sizes to significantly increase voxel size, resulting in concomitant increases in SNR and CNR. Lastly, to improve SNR in the body imaging model, we are currently seeking a surface coil to mirror our clinical 2H surface coil.

The first clinical use of deuterium MRI in humans was published in 2018 and has been validated for imaging at clinical magnetic field strengths (4, 23–26). Thus far, the deuterated substrate most extensively used has been [6,6-^2^H_2_]-glucose; however, this substrate may not be suitable for diabetic patients, those on systemic corticosteroids, or those on GLP-1 receptor agonists. Additionally, while d-glucose is less expensive than ^13^C-labeled isotopes, it is still quite costly. ^2^H_2_O has been widely used in clinical endocrinology metabolism studies and in cell kinetics research; hence, robust evidence supports its safety profile and lack of clinical toxicity (12). Proliferating cells, such as tumor cells, increase in volume to prepare for division, and require more water to maintain structure integrity. The mobility of intracellular (cytoplasmic) water has been shown to increase significantly in cancer cells, particularly in terms of rotational motion, while hydration water molecules remain unaffected (27). Moreover, water is crucial for a variety of biochemical reactions, serving as a solvent and a medium for transport, given their high rates of proliferation and metabolism, cancer cells often exhibit an increased water requirement (28). The water content in cancerous tissues can be nearly 30% higher compared to cells in the corresponding non-malignant tissue of origin (29).

In this study, we applied clinically applicable and relatively inexpensive ^2^H_2_O for systemic labeling to distinguish proliferating tumor from healthy tissues. In patients, to achieve similar TBW enrichment levels oral intake of approximately 250 ml ^2^H_2_O per day for 3–4 days prior to scan would be sufficient. Although systemic labeling with ^2^H_2_O results in deuterium incorporation into other macromolecules (13, 16, 30, 31), e.g., DNA and protein, they hardly contribute to the detectable dMRI signal due to their very short T_2_ relaxation time and much lower abundance. In contrast, ^2^H_2_O is the major contributor to dMRI signal during systemic labeling, which enables the use of dMRI to examine the biological differences between tumor tissue and normal tissue (13). Nevertheless, the ^2^H_2_O concentrations used in previous pre-clinical studies were relatively high, up to 20% v/v (13, 32), and above those with demonstrated clinical safety. Clinically, rapid ramp up of ^2^H_2_O labeling to high TBW concentrations can elicit dizziness/vertigo and nausea. Additionally, we previously investigated the influence of ^2^H_2_O concentrations and labeling duration on cancer cell proliferation rates and found the similarities between the control group and 5% ^2^H_2_O enrichment in TBW group, while higher levels of deuterium (> 20%) led to significantly reduced rates of cell proliferation (22), which could potentially affect the physiological functions of normal cells, although these effects are usually reversible (33). Hence, this study used low levels of ^2^H_2_O labeling in TBW (2% and 4% TBW enrichment) to optimize our labeling protocol in preparation for clinical translation. Finally, the volume of ^2^H_2_O that a cancer patient would have to imbibe to reach high TBW levels would be quite costly and would take too long to achieve safely without needing IV labeling, while timely diagnosis and staging are necessary for prompt initiation of cancer treatment. For example, for a 70-kg patient, 2% TBW enrichment would require an intake of approximately 850 ml of 100% ^2^H_2_O that can be performed safely over 3 days (20). To achieve 5% TBW, 2100 ml would need to be imbibed. Safely administering the latter via oral route, which is preferable for outpatient studies, would require longer labeling and more than double the isotope cost. Our pre-clinical study shows that 2% TBW provides sufficient, stable CNR, which supports our clinical implementation plans for 2% TBW goal.

To summarize, we report that tumors can be visualized via dMRI with adequate SNR even at relatively low clinically applicable ^2^H_2_O TBW enrichments that can be achieved in patients with small volumes of oral daily doses of ^2^H_2_O and several days duration (less than 1 week). The contrast between tumor and normal tissue is sufficient with short and low dose labeling, easing implementation in outpatient clinical settings for staging and subsequent assessment of therapeutic responses in cancer patients. Although the values of SNR, CNR and T/M may differ in the specific labeling data acquired at 3T compared with 7T, the pattern of change during the systemic labeling duration should be preserved across different magnetic field strengths. The study presented herein provides a basis for a cost-effective and well-tolerated use of ^2^H_2_O in clinical cancer imaging and should be further investigated.

## Data Availability

The original contributions presented in the study are included in the article/**Supplementary Material**. Further inquiries can be directed to the corresponding author.
