# Supplementary material for: Deuterium magnetic resonance imaging of tumors using low-dose systemic deuterated water labeling
Source: Front Oncol. 2026 Jan 12;15:1688968. doi: 10.3389/fonc.2025.1688968 (PMC12832314; doi:10.3389/fonc.2025.1688968)
Supplement: Supplementary file 1 [file DataSheet1.pdf]

MR Acquisition Parameters

MRI acquisitions were carried out on a Bruker Biospec, 7T MRI system on the Paravision 360 v3.3 imaging platform (Billerica, MA). For ^{1}H anatomical reference, a 3D, steady-state free precession scan (SSFP-FID) was acquired with the following acquisition parameters: FOV = 48×32×32 mm, matrix = 128×96×96, TE/TR/FA=2.0/6.5ms/10°, NEX = 1. T2-weighted (T2W), fast spin-echo scans were acquired in the coronal and axial views as well, with parameters shown in **Table S1**. Coronal scans were acquired in the same slice orientations as the ^{2}H FLASH sequences as denoted in the main text.

Table S1. T2-weighted image acquisition parameters with different coils and orientations

Scan	TE/TR (ms)	Matrix	FOV (mm)	Slices	Slice THK (mm)	NEX
T2W axial (leg)	28/2500	192×192	28×28	36	0.75	4
T2W coronal (body)	28/2500	256×192	48×32	35	0.80	2
T2W axial (body)	28/2500	192×192	32×32	27	0.08	2

MRI Co-registration Workflow

A schematic of the workflow for co-registering ^1H MR datasets is shown in **Figure S1**. T2-weighted spin echo scans offer the best tumor conspicuity to aid in drawing regions-of-interest (ROIs) but are more challenging to co-register accurately to the much lower SNR ^2H datasets. To address this, a ^1H 3D SSFP-FID dataset was acquired with identical geometry as the ^2H 3D FLASH scan, and subsequently co-registered to the lower resolution ^2H dataset, providing a high SNR, voxel matched ^1H dataset (Step 1, Figure S1). The T2-weighted spin echo scans were then co-registered to the transformed ^1H 3D dataset (steps 2A and 2B, Figure S1), providing T2-weighted datasets voxel-matched to the ^2H 3D FLASH dataset.

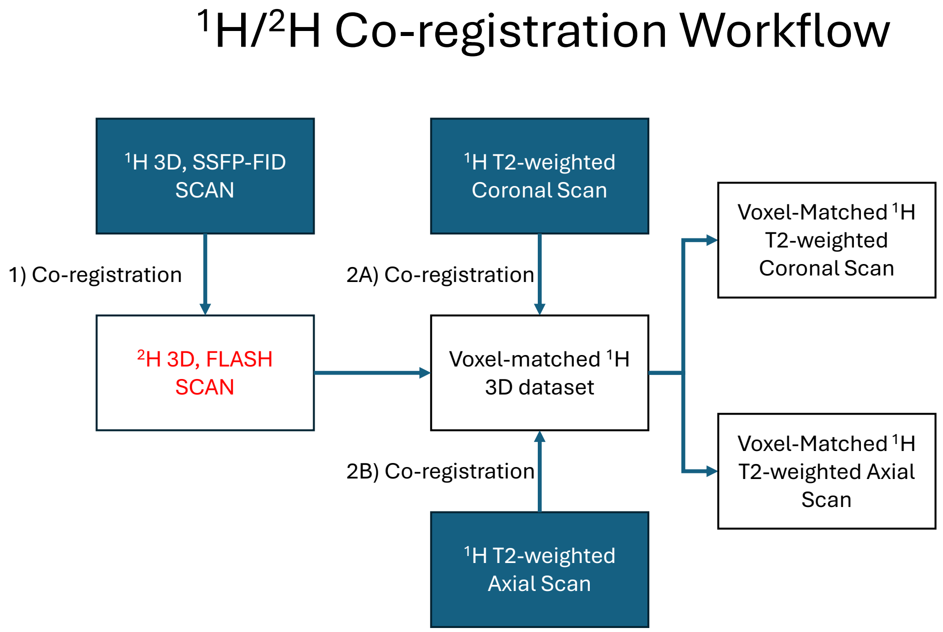

Figure S1. Co-registration of ^1H datasets to deuterium images. A geometrically-matched 3D, ^1H dataset is coregistered to the deuterium image (1), yielding a voxel-matched ^1H dataset, that can subsequently be used as a base image for co-registering the T2-weighted acquisitions (2A & 2B). In this manner, high-contrast T2-weighted ^1H images can be coregistered to the deuterium image.

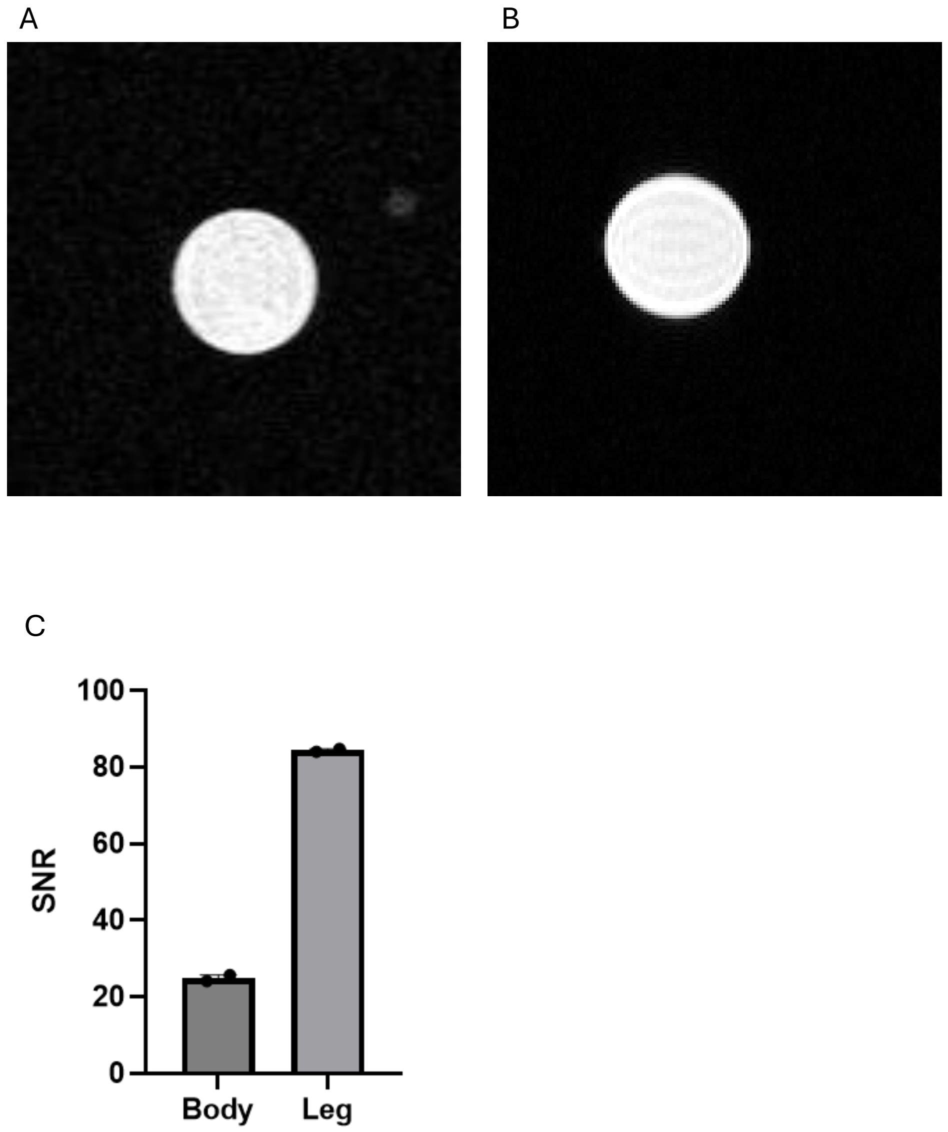

Figure S2. ^{2}H coil SNR comparisons normalized by volume (per mm^3) for deuterium imaging of a phantom containing 20% $^{2}H_2O$ in H_2O (v/v). Deuterium images were acquired using body volume coil (A) and leg volume coil (B) under identical scanning parameters: single-slice 2D FLASH with TE/TR/FA = 2.7/100ms/30°, matrix size = 128x128, FOV = 32×32 mm, slice thickness = 5mm, NEX = 32. (C) The SNR of leg coil was ~3.4-fold greater than that of body coil.
